# GRECCAR 8: impact on survival of the primary tumor resection in rectal cancer with unresectable synchronous metastasis: a randomized multicentre study

**DOI:** 10.1186/s12885-015-1060-0

**Published:** 2015-02-12

**Authors:** Eddy Cotte, Laurent Villeneuve, Guillaume Passot, Gilles Boschetti, Sylvie Bin-Dorel, Yves Francois, Olivier Glehen, The French Research Group of Rectal Cancer Surgery (GRECCAR)

**Affiliations:** 1 Department of Digestive Surgery, Hospices Civils de Lyon, Centre Hospitalier Lyon-Sud, Pierre-Bénite, France; 2 Université Lyon 1, EMR 3738, Lyon-Sud/Charles Mérieux Medical University, Oullins, France; 3 Hospices Civils de Lyon, Unité de Recherche Clinique, Pôle IMER, Lyon, France; 4 Department of Gastroenterology, Hospices Civils de Lyon, Centre Hospitalier Lyon-Sud, Pierre-Bénite, France

**Keywords:** Rectal cancer, Primary tumor resection, Unresectable metastasis, Palliative treatment, Survival, Quality of life

## Abstract

**Background:**

A majority of patients with rectal cancer and metastasis are not eligible to curative treatment because of an extensive and unresectable metastatic disease. Primary tumor resection is still debated in this situation. Rectal surgery treats or prevents the symptoms and avoids the risk of acute complications related to the primary tumor. Several studies on colorectal cancers seem to show interesting results in terms of survival in favor to the resection of the primary tumor. To date, no randomized trial or even a prospective study has assessed the impact of primary tumor resection on overall survival in patients with colorectal cancer with unresectable metastasis. All published studies were retrospective and included colon and rectal cancers. Rectal cancer is associated with specific problems related to the rectal surgery. Surgery is more complex, and may be source of more morbidity and postoperative functional dysfunctions (stoma, digestive, sexual, urinary) than colic surgery. On the other hand, symptoms related to the progression of rectal tumor are often very disabling: pain, rectal syndrome.

**Methods/Design:**

GRECCAR 8 is a multicentre randomized open-label controlled trial aimed to evaluate the impact on survival of the primary tumor resection in rectal cancer with unresectable synchronous metastasis. Patients must undergo upfront systemic chemotherapy for at least 4 courses before inclusion. Patients with progressive metastatic disease during upfront chemotherapy will be excluded from the study. Patients will be randomly assigned in a 1:1 ratio to Arm A: primary tumor resection followed by systemic chemotherapy versus Arm B: systemic chemotherapy alone. Primary endpoint will be overall survival measured from the date of randomization to the date of death or to the end of follow-up (2 years). Secondary endpoints will include progression-free survival, quality of life, toxicity of chemotherapy, response of the primary tumor and metastatic disease to chemotherapy, postoperative morbidity and mortality, rate of patient not eligible for postoperative chemotherapy (arm A), primary tumor related complications and rate of emergency surgery (arm B). The number of patients needed is 290.

**Trial registration:**

ClinicalTrial.gov: NCT02314182

## Background

Colorectal cancer (CRC) is the second most common cause of cancer deaths in Western countries and a significant public health issue with 38,000 new cases and 16,000 deaths annually in France [1]. Rectal cancer accounts for about one-third of colorectal cancers, with 14-18% of patients with synchronous metastatic disease (stage IV) at first presentation [2]. In stage IV disease, a curative strategy can only be proposed for selected patients when the primary and distant metastases are both resectable with 20-50% rates of long-term survival and cure after complete resection [1]. However, most (75-90%) metastases are unresectable leaning no curative option for these CRC with synchronous metastases. The treatment strategy will mostly be palliative, prolong survival with the best quality of life. In these cases, palliative chemotherapy can provide significant benefit in term of overall survival and quality of life [3,4]. Improvement in systemic chemotherapy have increased median survival from 9 months when only 5-fluorouracil (5-FU) was available to more than 24 months combination chemotherapy and biological agents [5-8]. But several studies suggested that primary tumor resection was probably beneficial for those patients.

### Impact on survival of primary tumor resection in patients with CRC and unresectable metastases

Several studies have assessed the impact of primary tumor resection for CRC with unresectable metastases [9-27]. None of the published studies were randomized. They were most often retrospective and reported by a single-centre. The major drawback in these studies was that surgery was offered to the patients with the best performance status and the preferred treatment for the other patients was chemotherapy alone. In addition, patients with extensive metastatic disease were more likely to be offered chemotherapy rather than surgery. In conclusion, there are some biases in these studies, and the results should be interpreted with caution. However, despite these limitations, a significant improvement of the median overall survival for the primary tumor resection group was observed in the majority of these studies.

Multivariable analysis found resection of the primary tumor to be an independent favorable prognostic factor [11,13-15,17,18,20,23,24,27,28]. The largest study was carried out by Venderbosh *et al.* [28]. They retrospectively analyzed two randomized trials, CAIRO and CAIRO II, which were originally designed to investigate the impact of chemotherapy on survival in patients with CRC and metastatic disease. The outcomes, including overall survival (OS) and progression-free survival (PFS), were analyzed on the basis of whether patients underwent resection of their primary lesion (n = 547) or not (n = 300) before chemotherapy. Patients who entered the CAIRO and CAIRO II study after resection of their primary tumor had longer OS (16.7 vs. 11.4 months and 20.7 vs. 13.4 respectively). PFS was also longer (marginally for CAIRO: 6.7 vs. 5.9 months and 10.5 vs. 7.8 for CAIRO II). Ferrand *et al.* published a study in 2013 using the data from the FFCD 9601 trial, which was designed to evaluate four lines of single agent chemotherapy regimen [29]. Out of the 216 patients with stage IV CRC, 156 patients had undergone resection of their primary tumor prior to entering the study. Both median OS (16.3 vs. 9.6, p > 0.0001) and PFS (5.1 vs. 2.9, p < 0.001) were significantly higher in the resection group. The multivariate analysis showed resection of the primary tumor to be the strongest independent prognostic factor for both OS and PFS. Although the analysis of the studies of Venderbosh *et al.* and Ferrand *et al.* appears straightforward, the observations can be explained by patient selection [28,29]. Indeed, in these two studies, patients were enrolled either at diagnosis, or after recovery from resection of the primary lesion. Therefore, neither the indication for resection, nor the morbidity and mortality associated with the surgery, were recorded. In fact, the denominator for the surgery cohort was patients who underwent surgery and recovered sufficiently to receive chemotherapy. We do not know how many patients underwent surgery and experienced complications which delayed chemotherapy, attenuated the course of chemotherapy, or rendered the administration of chemotherapy impossible. Furthermore, we do not know how many patients died directly as a result of primary tumor resection. A recent meta-analysis conducted by Stillwell *et al.,* but based on eight retrospective studies, has also shown an improvement in the survival of patients who underwent resection of their primary tumor compared to those treated with chemotherapy alone, with an estimated standardized median difference of six months (p < 0.001) [30]. And recently, two retrospectives studies using a propensity score analysis to limit biases also suggested the positive impact on survival of primary tumor resection in these situations (OS: 17.9 months vs 7.9 months, p > 0.0001 [31] and 13.8 vs 6.3 months, p = 0.0001 [32]).

### Impact on quality of life of primary tumor resection in patients with CRC and unresectable metastases

Quality of life is a critical aspect of palliative treatment. All previous studies evaluating the impact of primary tumor resection for patients with unresectable metastases have focused on survival and morbidity. Quality of life has never been specifically assessed, although it can be impacted by several parameters. Firstly, it may be impacted by the symptoms related to primary tumor and resulting complications. This is particularly true with rectal cancer, which is known for its disabling symptoms (pelvic pain, rectal syndrome, digestive hemorrhage, obstruction or abscess) when the primary tumor is progressing. Quality of life may also be impacted by postoperative morbidity after resection of the primary tumor or emergency surgery for complications related to the primary tumor. For rectal tumors - probably more than for colon tumors - morbidity and postoperative functional disorders (stoma, digestive, urinary or sexual disorders) are frequent and have an impact on quality of life. Chemotherapy tolerability, which may be different according to the presence or absence of the primary tumor, is another aspect of palliative treatment that may impact on quality of life.

### Complications related to unresected primary tumors

Unresected primary tumors can lead to tumor complaints and complications linked to the growth of the primary tumor, such as obstruction, perforation or bleeding. Emergency surgery was associated with high morbidity and even mortality [17,25,33-35]. The risk of local complications related to a tumor left in situ during chemotherapy varied from 8.5% to 30% and the highest risk was of obstruction (6-29%) [10,17,23,36-41]. These results need to be interpreted with caution, as they come from dated retrospective series that involved very few patients supported over long periods of time with heterogeneous chemotherapy regimens. In addition, many of these series included patients with for whom the primary tumor complicated primary tumors or who were symptomatic at first presentation [10,42,43]. With recent advances in systemic chemotherapy, the risks and benefits of an immediate or deferred surgical strategy have changed. In contrast to the response rates of 5-FU and leucovonrin of approximately 15%, combinations with modern chemotherapy, such as infusional fluorouracil/leucovorin with oxaliplatin or irinotecan have yielded response rates of 50% and disease control rates of 85% in prospective clinical trials [5,44]. Furthermore, adding the targeted agent bevacizumab and/or cetuximab to the above combinations has been shown to obtain a clinically significant improvement in response rates [4,6,7,45,46]. In the area of effective chemotherapy, the risk of primary tumor related complications and the subsequent need for emergency colectomy and/ or other primary tumor related interventions is low, less than 15% in most series. In the series reported by Muratore *et al.* and Poultsides *et al.* in which patients were mainly treated with effective chemotherapy (oxaliplatin, irinotecan, targeted agent) and had asymptomatic or uncomplicated primary tumors at presentation, the risk of complications was close to 10%, which can be explained by the response of the primary tumor to chemotherapy observed [47,48].

### Management of chemotherapy in the presence of the primary tumor

There are no specific studies in the literature that have evaluated the influence of the in situ primary tumor on chemotherapy tolerance and safety. In the EORTC phase III study, which showed an improvement in 3-year progression-free survival with perioperative FOLFOX based chemotherapy, compared to surgery alone in patients with initially resectable liver metastases (<4 metastases), for 34% of patients the primary tumor was in place at randomization and no increased toxicity was reported in these patients [49]. In several retrospective studies, no difference in chemotherapy toxicity was observed, regardless of whether or not the primary tumor was in place [15,16,50]. Bevacizumab has been associated with a 1% to 2% incidence of gastrointestinal perforation in prospective clinical trials [51,52]. In the study reported by Poultsides *et al.*, 48% of the patients received bevacizumab and only two of the five perforations observed (all at the site of the primary tumor) occurred during bevacizumab therapy; one patient experienced perforation 6 months after the final administration of bevacizumab, whereas two were naïve to this agent [48]. Although the small number of patients who developed this complication precludes drawing any definitive conclusions, bevacizumab did not appear to increase the rate of perforation. One other aspect is the efficacy of the chemotherapy in presence of the primary tumor. Whether or not the primary tumor has an impact on chemotherapy efficacy is debated. A recent retrospective study on 409 patients with metastatic colorectal cancer treated by chemotherapy suggested that bevacizumab improved overall survival only for patient who were operated before chemotherapy for primary tumor resection [53]. For patients without K-RAS mutation, anti-EGFR antibodies are also a possibility, although no study has yet examined the effect of these antibodies in patients with a metastatic CRC and a primary tumor in place [4].

### Morbidity of primary tumor resection in the setting of unresectable metastasis

For patients operated for their primary tumor as part of their initial management, the question of the potential extra-risk of postoperative morbidity associated with the resection of the tumor in metastatic setting should be addressed. Several studies have suggested that resection of the primary tumor in the presence of metastatic disease is associated with high postoperative morbidity and mortality rates [17,28]. One study by Stelzner *et al.* reported that 15 out of 128 patients (11.7%) patients died within 30 days of surgery [17]. However, in this study many of these patients were symptomatic and underwent emergency surgery. The same series found a 27.8% mortality rate in patients who underwent emergency surgery compared to a 7.3% mortality rate for elective procedures (p = 0.002). The high postoperative mortality rate of 4.6% reported by Scoggins *et al.* included patients who were symptomatic at the time of resection and the patients who died after surgery were found to have severe carcinomatosis [9]. These mortality rates were higher than those found in a recently-published meta-analysis in which collectively, perioperative mortality was 1.7% (95% CI 0.7%-3.9%) [30]. This lower mortality rate can be accounted for by the fact that most patients within this meta-analysis were asymptomatic and were managed electively. In this meta-analysis, postoperative morbidity occurred in 68 of 299 patients for a pool proportion of 23% (95% CI 18.5-21.8). The most frequent complication was wound infection which could be managed conservatively; however, in some instances, a major complication arose requiring additional surgery. Anastomotic leakage, occurring in 1.7% of patients (5/299 patients) in the series included, is more commonly a significant complication of rectal cancer resection. It often leads to sepsis, significantly prolongs hospital stays and delays or even precludes the administration of chemotherapy [30]. With modern management (perioperative immunonutrition, laparoscopic resection, enhanced recovery after surgery), the morbidity of an elective rectal surgery for an asymptomatic patient with unresectable metastasis is probably not so high as it was described in the literature in the past.

### Patient selection for primary tumor resection

Surgery should be avoided in patients with rapidly progressing tumors [54]. Progression during preoperative chemotherapy should be regarded as a biological marker for poor prognosis and an indication for administering second-line chemotherapy before considering surgery [49]. Progression during first-line chemotherapy occurs in about 5-10% of patients [55-57]. Stelzner *et al.* conducted a multivariable analysis, which showed that the resection of the primary tumor in CRC with unresectable metastases was a predictor of prolonged survival, and that chemotherapy was the only treatment-related factor associated with prolonged survival on an intention-to-treat basis [17]. They therefore concluded that chemotherapy should be the first treatment step for these patients, selecting a group of patients who might benefit from a deferred resection of the primary tumor. In another study, they demonstrated the feasibility of this option in patients with resectable rectal cancer and unresectable metastases [58]. In a small cohort of 22 patients, 7 patients without tumoral progression under first-line chemotherapy finally underwent resection of their primary tumor and showed a higher median overall survival than patients without resection (27.2 months vs. 12.4 months, p = 0.017). There were no post-operative deaths in this study. In case of palliative treatment where the option of complex surgery on a primary rectal tumor is envisaged, chemotherapy should be the first treatment and surgery should only be proposed when there is no progression during preoperative chemotherapy. Patients with a poor prognosis due to progressing disease are thereby spared the risks of major rectal surgery with unnecessary surgical complications.

## Methods/Design

### Protocol overview

**Figure 1:**
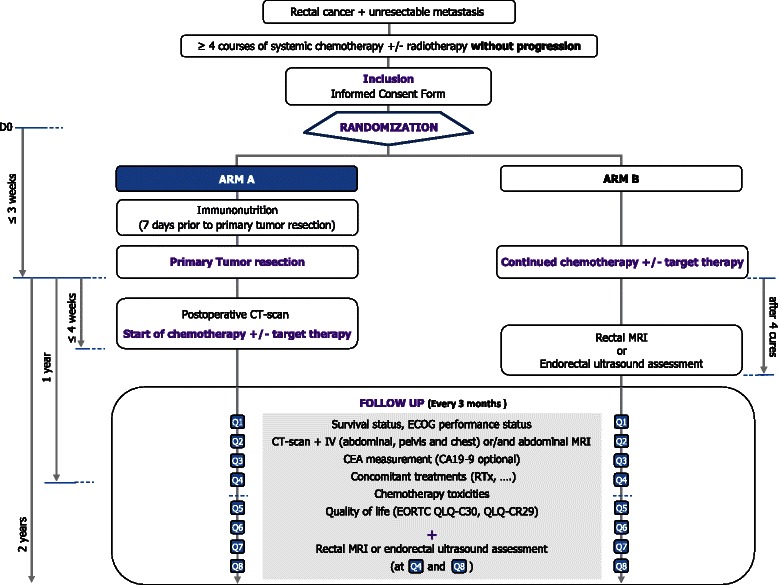
**GRECCAR 8-Flow chart.**

This study is a multicentre randomized open-label controlled trial aimed to evaluate the impact on survival of the primary tumor resection in rectal cancer with unresectable synchronous metastasis (Figure 1- Flow chart study). Patients will be randomly assigned in a 1:1 ratio to Arm A: primary tumor resection followed by systemic chemotherapy versus Arm B: systemic chemotherapy alone.

### Participants

The institutional sponsor is the HCL-DRC (Hospices Civils de Lyon- Département de la Recherche Clinique). This study is supported by the French Research Group of Rectal Cancer Surgery (GRECCAR) and the French Federation of Surgical Research (FRENCH). Patients will be included from 25 centres in France (see the list of participating centres in acknowledgments section). All patients must fulfil the following criteria: resectable rectal cancer with unresectable metastasis and no progression during upfront chemotherapy. The complete inclusion and exclusion criteria are given Table 1.

**Table 1:** **Inclusion and exclusion criteria**

Inclusion criteria	Exclusion criteria
• Non-complicated primary tumor (i.e. tumor without obstruction, bleeding, abscess or perforation requiring emergency surgery and/or contra-indicating first-line chemotherapy)	• Rectal tumor operated before inclusion
• Unresectable synchronous metastases	• Resectable metastases
• Rectal adenocarcinoma (<15 cm from the anal verge) with few or no symptoms and unresectable metastasis (assessed by the investigator) unsuitable for curative treatment	• Symptoms related to the rectal tumor requiring first intention rectal surgery (appreciated by investigator)
• No known unresectable primary tumor (with clear margin > 1 mm) on CT-scan and MRI	• Contra-indication for surgery
• No disease progression under chemotherapy (for at least 4 cycles)	• Complicated (obstruction, bleeding, abscess, perforation) primary tumor requiring emergency surgery and/or contra-indicating first line-chemotherapy
• Assessment of KRAS status before randomization (wild type or mutated)	
• ECOG performance status 0-1	• Non-resectable primary tumor (with wild margin)
• Life expectancy without cancer >2 years	• Under nutrition (albumin < 30 g/l)
• White blood cell count ≥ 3 × 10^9^/L, with neutrophils ≥ 1,5 × 10^9^/L, platelet count ≥ 100 × 10^9^/L, hemoglobin ≥ 9 g/dL (5,6 mmol/l)	• Peritoneal carcinomatosis
• Total bilirubin ≤ 1.5 x ULN (upper limit of normal), ASAT and ALAT ≤ 2.5 × ULN, alkaline phosphatase ≤ 1.5 × ULN, serum creatinine ≤ 1.5 × ULN	• Disease progression under chemotherapy (RECIST 1.1 criteria)
• Age ≥ 18 years ≤ 75 years	• Known hypersensitivity reaction or specific contraindications to any of the components of study treatments
• Patients with childbearing potential should use effective contraception during the study and the following 6 months	• Clinically relevant coronary artery disease or history of myocardial infarction in the last 12 months, or high risk of uncontrolled arrhythmia
• Covered by a Health System where applicable, and/or in compliance with the recommendations of the national laws in force relating to biomedical research	• Pregnancy (absence to be confirmed by ß-hCG test) or breast-feeding
• Signed written informed consent obtained prior to any study-specific screening procedure	• Previous malignancy in the last 5 years

### Randomization

After upfront chemotherapy without tumoral progression, if the patient has given informed, written consent and meets inclusion criteria, he will be randomized, using an interactive Web response system. Randomization will be balanced and stratified by investigating centre and localisation of the primary tumor (high versus middle/low rectal cancer).

### Treatments

#### Chemotherapy

Patients will receive systemic chemotherapy according to standard local practices. The choice of the different chemotherapy regimens and the management of the medical treatment are left to the investigator’s appreciation throughout the patient’s treatment. The investigator will be able to choose the type of chemotherapy considered as the most effective in light of current scientific data and the patient’s general health or nutritional status after discussion and validation by each MDT (Multidisciplinary Team). The most common combinations are oxaloplatin or irinotecan plus capecitabine or 5-FU with or without bevacizumab. In cases of K-RAS wild-type tumors, anti-epidermal growth factor receptor (EGFR) antibodies panitumumad and cetuxiamb can be used.

Before inclusion, patients will receive upfront chemotherapy for at least four cycles. If patients are already undergoing systematic chemotherapy, inclusion is possible if no progression has been observed during the last four courses of chemotherapy. If the patient has received no treatment at all, they must receive at least four courses of systemic chemotherapy in order to assess disease control under chemotherapy. If there is no progression during the chemotherapy, the patient can be included. In case of disease progression, the chemotherapy regimen must be switched, and patient can be included if there is no disease progression after four courses of chemotherapy. If eligible, patients can be included in another trial of first line metastatic CRC.

After randomization in Arm A, chemotherapy will start within 4 weeks after surgery. Bevacizumab should not be administered during the six weeks before and after surgery. In Arm B, chemotherapy will start within three weeks after randomization. If complications occur, emergency surgery can be performed according to the local practices of each investigator center.

#### Surgical resection

Primary tumor resection will be performed within 3 weeks after randomization. Immunonutrition will be given seven days prior to primary tumor resection following the 2011 HAS labeled French clinical guidelines. Mechanical bowel preparation will be performed before surgery according to the local practices of each investigator center. Primary tumor resection will be performed by laparoscopy (recommended) or by laparotomy according to the local practices of each investigator centre. The type of resection (coloanal or colorectal anastomosis, abdominoperineal resection, delayed anastomosis, …) is let at the investigator’s discretion but must fulfill with the oncological quality criteria.

#### Radiotherapy

Radiotherapy or chemoradiotherapy is authorised in both arms if necessary, for downstaging in Arm A before surgery or for symptomatic control in Arm B.

### Outcomes and assessments

#### Primary outcome

Overall survival will be measured from the date of randomization to the date of death or to the end of follow-up (2 years).

#### Secondary outcomes

Progression-free survival, quality of life, toxicity of chemotherapy, response of the primary tumor and metastatic disease to chemotherapy, time to disease progression, rate of secondary curative resection, postoperative morbidity and mortality, rate of patient not eligible for postoperative chemotherapy (arm A), primary tumor related complications and rate of emergency surgery with post-operative morbidity and mortality associated (arm B).

#### Pre-therapeutic work-up

Patients eligible for the study will be seen in clinics following upfront chemotherapy to check the inclusion and exclusion criteria. The patient will be required to give written informed consent to participate to the study before any non-routine screening tests or evaluations are conducted. The following assessments should be performed: Performance Status, quality of life (EORTC QLQ-C30, QLQ-CR29), colonoscopy with biopsy, KRAS mutation assessment, thoraco-abdominopelvic CT scan, rectal MRI or endorectal ultrasonography, laboratory exams: serum CEA, CA19-9; haemoglobin, leukocytes, neutrophils, platelets, glycemia, AST, ALT, LDH, total bilirubin, alkalin phosphatase, serum albumin, total protein, plasmatic APTT, PT and INR; creatininemia and creatinine clearance.

#### Follow-up

The postoperative morbidity and mortality will be assessed. Postoperative morbidity is defined as surgical or medical complications that occur within 30 days after surgical intervention. The evaluation of postoperative morbidity and mortality will be assessed in the primary tumor resection arm (arm A) and in the chemotherapy arm (arm B) for patients who require emergency surgery. The post-operative complications will be evaluated according to the Clavien-Dindo Classification of Surgical Complication and graded 0 to V [59]. Chemotherapy toxicity will be graded according to the National Cancer Institute Common Toxicity Criteria for Adverse Events (NCI-CTC-AE V4.0) in both treatment arms.

The response to systemic chemotherapy of the primary rectal cancer will be evaluated after 4 courses of chemotherapy, and at one and two year after randomization in the chemotherapy arm B using MRI or endorectal ultrasound (maximal sizes and tumoral volume evaluated by MRI). The response rate of the metastatic disease will be evaluated in both treatment arms after 4 cycles of chemotherapy and then every three months by CT scan and analyzed using the RECIST 1.1 criteria [60]. Time to disease progression is defined as the lapse of time between the date of randomization and the first date of progression (clinical or imaging) of the metastatic disease in both treatment arms, or of the primary rectal tumor in the chemotherapy arm (arm B). The rate of secondary curative resection will be assessed in both treatment arms and will concern resection of both the primary tumor and the metastatic disease. Those rates will be estimated at 12 months and for the whole follow-up period. After surgery, progression will be defined as an obvious appearance of a recurrence on imaging (CT and/or MRI and/or PET) or proven by biopsy. Primary tumor complications such as obstruction, bleeding, abscess and perforation will be collected in the chemotherapy arm (arm B). The rate of primary tumor related complications will be assessed at 1 month after the administration of the first cycle of chemotherapy and then every 3 months during follow-up (clinical and using imaging). The rate of emergency surgery related to the rectal cancer will be assessed in the chemotherapy control arm (arm B).

For all patients, follow-up assessment will be performed until progression and/or death. For the purposes of the study, overall survival and progression-free survival will be assessed at 2 years. For patients included in the present trial, the follow-up will be systematic and performed throughout the two-year period according to good clinical practice.

Every 3 months during the 2 years, the following investigations will be performed: clinical assessment (performance status); tumor assessment by CT-scan with contrast enhancement or MRI if CT scan is impossible (kidney failure, allergy to iodine) or insufficient to characterize lesions; CEA measurement (CA19-9 optional); survival status; additional cancer therapy; assessments of chemotherapy toxicities and quality of life (EORTC QLQ-C30, QLQ-CR29).

### Sample size and statistical considerations

According to the three retrospective analyses from randomized studies (FFCD 6901, CAIRO and CAIRO II), a 15% improvement in overall survival at 2 years is expected in the surgical group. Taking the worst-case hypothesis (i.e. high survival in the control arm = 25%) and in order to obtain 80% power with a two-side alpha level of 5%, with a follow-up period of 2 years, 138 patients are required per arm. To allow for potential drop-outs, 145 patients will be included per arm. A total of 290 patients will be included. Each patient will participate and be followed up for 2 years from their randomization date. The recruitment period is planned for 36 months, in 25 centres. The total duration of the study will be 5 years.

### Ethical considerations, information giving and written informed consent

The study protocol was approved by the Institutional Review Board: the Sud Est IV ethics committee on the September 23^rd^, 2014 and by the French National Agency for Medicine and Health Products Safety (ANSM) on the June 3^rd^, 2014. The Ethical approval was given for all participating centres. The research carried out will be on accordance with the Helsinki Declaration and ICH GCP Guidelines. The study complies with the principles of the Data Protection Act. This study is supported by a grant from the French ministry of health (PHRC-K13-069). The study has been registered on the ClinicalTrial.gov website under the identification number NCT02314182.

For each patient recruited into the study, written informed consent is essential prior to inclusion into the study after extensive information about the intent of the study, the study regimen, potential associated risks and side effects as well as potential alternative therapies. The investigator will not undertake any diagnostic measures specifically required for the clinical trial until valid consent has been obtained.

## Discussion

Exclusive systemic chemotherapy is most often used to treat patient with rectal cancer and unresectable metastasis. During this treatment, patients may require emergency surgery to treat complications related to the primary (obstruction, bleeding or perforation). Such unplanned operations are associated with higher operative morbidity and mortality to scheduled procedures for stage IV disease. Many surgeons have advocated resection of the primary mainly to avoid these complications however this surgery may delay the start of chemotherapy. Some studies reported that resection of the primary in case of unresectable metastatic disease prolonged survival. These studies were non-randomized and the majority were single-centre and retrospective. The major drawback of these non-randomized studies was that the patients selected for surgery were those with a better performance status and better prognosis. Furthermore, the impact of primary tumor resection on quality of life has not been assessed. All published studies included colon and rectal cancers, but the issues are different for these two localizations. Surgery for rectal cancer seems more complex and may result in increased morbidity and postoperative functional disorders (stoma, digestive, sexual, urinary) compared to colonic surgery. On the other hand, symptoms related to the progression of rectal tumor are often severely disabling (pain, rectal syndrome) and difficult to control. There is currently a French multicentre study underway named CLIMAT- PRODIGE 30 (Pr M. Karoui, Paris, Pitié-Sapétrière Hospital) studying this specific problem of palliative resection in colon cancer (rectal cancers are excluded). Whether or not resection of the primary tumor in palliative care is beneficial remains a clinical dilemma. To date, no randomized trial has assessed the impact of primary tumor resection on overall survival and quality of life in patients with CRC and unresectable metastases. GRECCAR 8 is a dedicated prospective randomized study looking into the specific problem of rectal cancer to evaluate the impact of primary tumor resection in rectal cancer with unresectable metastases.

## Abbreviations

CRC: colorectal cancer
5-FU: 5-fluoro-uracil
GRECCAR: French Research Group of Rectal Cancer Surgery
FRENCH: French Federation of Surgical Research
MDT: Multidisciplinary team
EGFR: Epidermal growth factor receptor
HAS: High authority for health
EORTC: European Organisation for Research and Treatment of Cancer
CT: Computed tomography
MRI: Magnetic resonance imaging
CEA: Carcinoembryogenic antigen
CA19-9: Carbohydrate antigen 19–9
AST: Aspartate aminotransferase
ALT: Alanine aminotransferase
LDH: Lactate deshydrogenase
APTT: Activated partial thromboplastin time
PT: Prothrombin time
INR: International normalized ratio
NCI-CTC-AE: National Cancer Institute Common Toxicity Criteria for Adverse Events
RECIST: Response evaluation criteria in solid tumors
PET: Positron emission tomography
ANSM: French National Agency for Medicine and Health Products Safety
